# Who Pays? Coverage Challenges for Cardiovascular Genetic Testing in U.S. Patients

**DOI:** 10.3389/fcvm.2016.00014

**Published:** 2016-05-31

**Authors:** Katherine G. Spoonamore, Nicole M. Johnson

**Affiliations:** ^1^Department of Medicine, Krannert Institute of Cardiology, Indiana University School of Medicine, Indianapolis, IN, USA; ^2^Invitae Corporation, San Francisco, CA, USA

**Keywords:** genetic testing, insurance coverage, cardiovascular genetics, preventative care, access barriers, cascade testing

## Abstract

Inherited cardiovascular (CV) conditions are common, and comprehensive care of affected families often involves genetic testing. When the clinical presentations of these conditions overlap, genetic testing may clarify diagnoses, etiologies, and treatments in symptomatic individuals and facilitate the identification of asymptomatic, at-risk relatives, allowing for often life-saving preventative care. Although some professional society guidelines on inherited cardiac conditions include genetic testing recommendations, they quickly become outdated owing to the rapid expansion and use of such testing. Currently, these guidelines primarily discuss the benefits of targeted genetic testing for identifying at-risk relatives. Although most insurance policies acknowledge the benefit and the necessity of this testing, many exclude coverage for testing altogether or are vague about coverage for testing in probands, which is imperative if clinicians are to have the best chance of accurately identifying pathogenic variant(s) in a family. In response to uncertainties about coverage, many commercial CV genetic testing laboratories have shouldered the burden of working directly with commercial payers and protecting patients/institutions from out-of-pocket costs. As a result, many clinicians are unaware that payer coverage policies may not match professional recommendations for CV genetic testing. This conundrum has left patients, clinicians, payers, and laboratories at an impasse when determining the best path forward for meaningful and sustainable testing. Herein, we discuss the need for all involved parties to recognize their common goals in this process, which should motivate collaboration in changing existing frameworks and creating more sustainable access to genetic information for families with inherited CV conditions.

## Introduction

Inherited cardiovascular (CV) conditions include arrhythmias, cardiomyopathies, aortopathies, and dyslipidemias. These conditions affect more than 1 in 200 individuals, and several of them have considerable phenotypic overlap. Therefore, comprehensive care of affected patients and families often involves multi-gene panel-based genetic testing, which can clarify diagnoses and etiologies. Since becoming available in the early to mid-2000s, panel-based CV genetic tests have seen widening clinical adoption. The range of conditions covered by current commercial CV genetic testing and the number of genes included in analyses have also expanded exponentially.

Advances in CV diagnostics have spurred changes for insurance companies, genetic testing laboratories, and professional cardiology societies, which have created coverage policies, developed expanded panel-based tests, and formulated care guidelines, respectively. However, the question of who pays for CV genetic testing is ongoing and relates to both the applicability of genetic testing in probands and at-risk relatives and the need for sustainability in laboratory services and payer policies.

Herein, we review the current status of genetic testing guidelines for inherited CV conditions and the roles of payers and genetic testing laboratories in providing access to testing for affected families. We also discuss the inconsistencies among clinical approaches, professional society guidelines, payer policies, and laboratory practices that have influenced this access. Finally, we highlight the shared goals of all stakeholders and discuss how these overlapping interests are a starting point on the path to sustainable, accessible genetic testing for patients with inherited CV conditions.

## History of CV Genetic Testing

Although genotype–phenotype correlations remain in their infancy in CV genetics, genetic test results can have key impacts on patient care and management by clarifying clinical presentation and etiology, aiding decision-making about surgical procedures, and guiding medication selection and surveillance strategies. For example, long QT syndrome subtyping (for types 1, 2, and 3) provides some indication of both responsiveness to certain treatments and the presence of higher-risk situations that may trigger cardiac events. In cardiac hypertrophy, genetic testing can identify underlying causal conditions (e.g., Fabry disease, Danon disease, Pompe disease, transthyretin amyloidosis). Alfares et al. (1) found that 3% of hypertrophic cardiomyopathy (HCM) patients who underwent genetic testing had an undetected syndromic disease that presented an opportunity for more effective treatment (e.g., enzyme replacement therapy in Fabry disease). Earlier clarification of cardiomyopathy etiology through genetic testing in children, in which metabolic causes are much more frequent, can also improve treatment outcomes. When cardiomyopathies are associated with conduction disease or higher arrhythmogenic potential, the increased likelihood of changes in certain genes (e.g., *SCN5A*, *LMNA*) warrant closer surveillance and specific intervention from a cardiac electrophysiologist and a heart failure/cardiomyopathy specialist (2). Moreover, in the case of overlapping aortopathies (Marfan syndrome versus Loeys–Dietz syndrome), genetic test results can guide the timing of surgical intervention, which differs based on the etiology of aortic disease (3). The risks for recurring aortic aneurysms/dissection, the most vulnerable portions of the aorta, and the involvement of additional vasculature also vary according to etiology; therefore, genetic testing can guide the choice of imaging method and frequency of ongoing surveillance.

Until recently, long turnaround times – typically 8–12 weeks – could be expected for CV genetic tests. Therefore, the results have generally been less routinely useful for planning immediate patient care. Furthermore, CV genetic test results may not have direct management implications for the individual tested. Often, the primary benefit of CV genetic testing comes in uncovering pathogenic variants in probands that can then be used for targeted genetic testing to identify at-risk family members (cascade screening) and plan their surveillance and, equally important, reduce risk in family members to the population baseline when they test negative for variants. Early diagnosis of hereditary CV conditions improves outcomes; therefore, early identification of at-risk family members improves outcomes as well.

Historically, CV genetic testing has been covered only sporadically by insurance and has been cost prohibitive for patients. The ability to direct family cardiac screening is valuable for both patients and payers, but this reason alone is not always a convincing argument for why payers should cover testing for probands. For example, Medicare specifically prohibits the genetic testing of both affected patients and asymptomatic at-risk family members, if the test will benefit individuals other than Medicare patients themselves.

## Professional Guidelines for CV Genetic Testing

Practice guidelines drafted by professional cardiology and genetics societies aim to provide patient care recommendations (evidence-based, when possible) that lead to the best clinical outcomes. The guidelines for the inherited arrhythmias and cardiomyopathies currently address genetic testing most thoroughly. These guidelines were published in 2009 by the Heart Failure Society of America (4) and in 2011 by the American College of Cardiology Foundation/American Heart Association (ACCF/AHA) (5) and the Heart Rhythm Society/European Heart Rhythm Association (HRS/EHRA) (2).

Genetic testing is a class I recommendation (“is recommended”) in probands for just 5 of the 13 conditions covered in the HRS/EHRA document, including individuals with strong clinical suspicion of long QT syndrome, catecholaminergic polymorphic ventricular tachycardia, HCM, and dilated cardiomyopathy in the presence of conduction disease or a family history of premature unexpected death, as well as individuals who have survived an out-of-hospital cardiac arrest when a specific channelopathy or cardiomyopathy is suspected. However, cascade testing for a pathogenic variant, previously identified in a family proband, is a class I recommendation for all but 1 of the 13 conditions included.

The available professional guidelines are sometimes inconsistent. For example, unlike the HRS/EHRA statement described above, the ACCF/AHA guidelines for HCM recommend genetic testing only for probands with atypical presentations that raise suspicion of an underlying syndromic etiology. For all other individuals with HCM, the guidelines classify genetic testing as a class II recommendation (“is reasonable”), specifically to facilitate the identification of at-risk family members (5).

Unlike the National Comprehensive Cancer Network guidelines for oncology, which are updated annually to provide recommendations for genetic testing, the guidelines provided by professional societies in CV medicine are updated too infrequently to serve as comprehensive recommendations for patient management. In addition, the National Comprehensive Cancer Network guidelines are highly specific about the genes to test and how the results of testing will influence management and surveillance of the proband undergoing testing. A lack of available clinical data on the use of genetic testing to improve long-term outcomes in patients with inherited CV conditions means that much of the professional guidance for cardiac genetic testing is based on expert opinion and experience rather than accumulated evidence. As such, the NCCN guidelines are closely followed by many major payers, unlike the current cardiology recommendations, which are rarely consistent with insurance coverage policies and may be considered insufficient by payers for determining which tests and which individuals to cover in affected families.

The American College of Medical Genetics and Genomics has published a “must-report” guideline related to clinically useful pathogenic test results of whole-exome or whole-genome analysis (6). The guideline specifies 56 genes for which findings are important for all patients to know and should be conveyed by clinicians even if they are secondary to a patient’s original indication for genetic analysis. This guideline underscores the value of genetic information for clinicians engaged in patient care and their desire to use genetic test results to guide the care they provide. Thirty-one of these genes are related to cardiac conditions; however, commercial payers do not cover genetic testing for some of these genes, even in probands suspected of having the condition.

## Payer Policies

Payer policies are driven by the goal of providing quality health care to all clients in a sustainable, cost-effective way. The downstream cost savings of initiating appropriate genetic testing in a family proband with cardiomyopathy followed by targeted genetic testing in related family members are considerable (1, 7, 8). These savings occur when relatives who did not inherit a known familial pathogenic variant can be released from further cardiac surveillance, and the testing and intervention recommendations for at-risk family members can be refined and optimized. However, evidence of these cost savings has not yet translated to wider payer coverage for genetic testing in probands.

Coverage policies for CV genetic testing are inconsistent among payers. When policies do include panel testing, different payers sometimes cover testing for different genes for the same condition. Because professional guidelines do not offer up-to-date, gene-specific, evidence-based guidance, it is unclear who is selecting the genes to be covered and what information is guiding or informing the selections.

For clinicians and families, these inconsistencies impede efficient decision-making and delivery of care. Written policies on medical necessity for specific CV conditions are often unavailable, which means that clinicians and patients have no assurance that genetic testing will be authorized or covered. This lack of specific documentation exists even for Medicare/Medicaid policies, in which coverage details for non-oncology genetic testing rarely exist and, when present, are tied to medical necessity. Medical necessity often remains undetermined by payers until a claim is submitted, and the expectations of patients and clinicians about the medical necessity of testing often differ significantly from the definitions adhered to by payers.

Even when CV genetic testing is covered, clinical decisions are further complicated by the extent of coverage provided. In many cases, payers cover only testing deemed medically necessary for the individual covered. Familial probands must undergo genetic testing to determine the underlying genetic cause of an inherited CV condition before cascade testing can begin. Until then, at-risk family members whose genetic testing is medically necessary and covered by insurance cannot obtain authorization for testing. Coverage denials based on medical necessity in probands create obstacles for both determining the underlying genetic cause of a familial CV condition and allowing at-risk family members to take advantage of genetic testing that is covered under their policies. Coverage/no-coverage combinations within affected families can become ongoing catch-22s in the management of life-threatening health conditions.

To the best of our knowledge, only one policy, from the commercial payer Aetna (9), successfully navigates the murky waters of proband/at-risk relative coverage for genetic testing. This policy states that the payer will cover *oncologic* genetic testing for a non-member familial proband whose own insurance has denied coverage, if the results are needed to pursue the medically necessary targeted testing of a covered at-risk family member. How often this coverage clause is used or honored, and what mechanism the payer has established to extend such coverage are unknown, but this example may be a model for consideration in CV genetics, in particular, because the primary benefit of most CV genetic tests is the identification of at-risk relatives.

## Commercial Laboratory Billing Policies

Genetic testing laboratories aim to provide quality, maximally accessible genetic testing to patients and clinicians. Costs and payment processes for genetic testing are generally dictated by the method in which tests are ordered and billed. Institutional billing, in which an institution (hospital) pays the laboratory performing the test and then bills and collects the payment from both the patient’s insurance and the patient, is the only option for some clinicians. Other institutions do not allow clinicians to use institutional billing, and instead, require them to work with laboratories that can bill payers directly.

**Figure 1:**
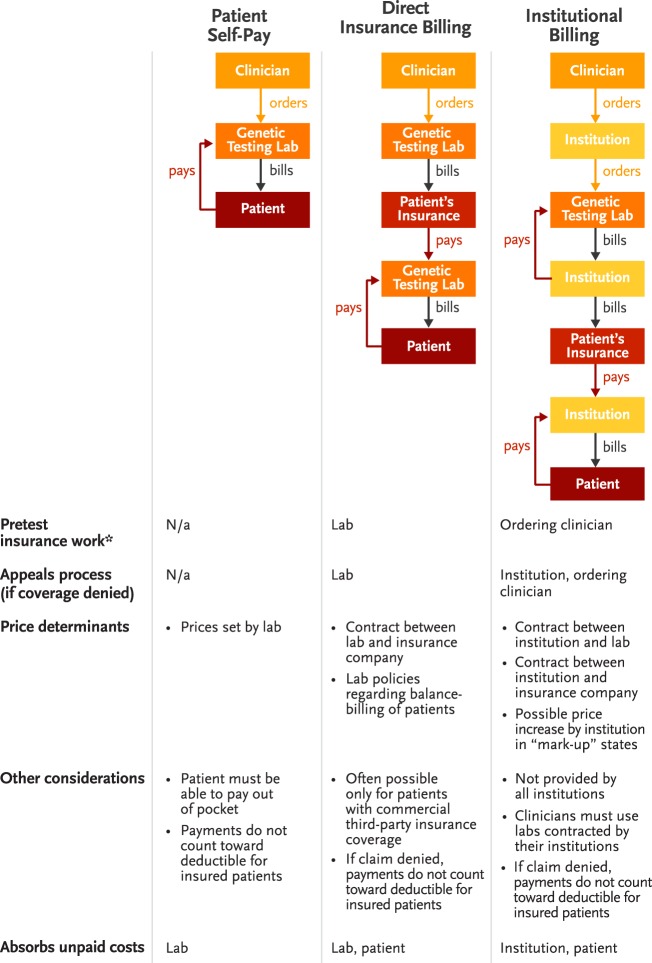
**Billing and payment pathways for genetic testing**. *Pretest insurance work includes benefits investigation, pre-certification, prior authorization, and discussion of costs with patients.

Laboratories that cannot bill insurers directly may require that all orders be handled by an institutional billing process at the clinician’s facility. To cover the costs associated with billing and collecting from both patients and payers, institutions in so-called mark-up states may charge more for testing. Figure 1 demonstrates the complexity of current billing processes for genetic testing.

Many commercial laboratories that bill patient insurance companies directly also devote customer service resources to payment planning or cost reductions for qualifying patients who sometimes bear significant out-of-pocket costs despite having insurance coverage. However, these additional services may be unavailable to patients with non-commercial insurance and are advantageous only to clinicians who can send samples directly to testing laboratories.

Cost often emerges as a key factor for patients in deciding whether to pursue testing recommended by their clinicians. Having laboratories take responsibility for billing and coverage/cost determination has increased both patient access and clinician utilization of CV genetic testing but has not necessarily improved insurance coverage for these tests. Furthermore, taking these processes out of the hands of clinicians has created a situation in which genetic testing stakeholders do not always realize that treatment decisions, professional guideline recommendations, and payer coverage policies are misaligned.

## Challenges in the CV Clinic

The disconnect between practice guidelines and coverage policies presents barriers to the timely and effective provision of care to patients with inherited CV disorders. Patient confusion can arise when testing that clinicians call “recommended” is considered “experimental” or “investigational” by payers. With vastly different payer policies or no clear policy to rely on, clinicians have difficultly determining whether patients can proceed with genetic testing and, if so, when testing can take place and what out-of-pocket expenses will be incurred (e.g., which tests are covered and which are not and which billing process – the institution’s or the laboratory’s – will yield the lowest out-of-pocket expense). The time required to find answers to these questions could be better spent counseling patients, and the delays in testing that occur while insurance policies are being clarified can create added concern for patients facing potentially serious diagnoses.

Furthermore, clinicians are inadequately trained to advise patients about the implications of various billing policies, and their lack of expertise may introduce legal liabilities. Few clinicians can differentiate among a pre-verification, a pre-determination, and a pre-authorization, for example, and even if they can, obtaining these clearances from payers often does not guarantee coverage. Discussions about expense are appropriate and necessary in decisions about patient care and management. However, compared with other routinely ordered medical tests (e.g., echocardiogram, electrocardiogram, magnetic resonance imaging) in cardiology clinics, orders for genetic testing frequently require clinicians to take a more prominent financial/insurance counseling role because uncertainties about coverage put cost at the center of diagnosis and treatment decisions.

In some cases, clinicians may alter the genetic testing strategy in a family based on the type of insurance coverage available for the required test – for example, selecting a different relative with a better insurance situation for testing. Gathering and assessing all of the necessary documentation to make decisions about testing logistics has the potential to be a complicated process that prevents some patients from receiving recommended and appropriate CV genetic testing in a timely manner.

## Path Forward

**Figure 2:**
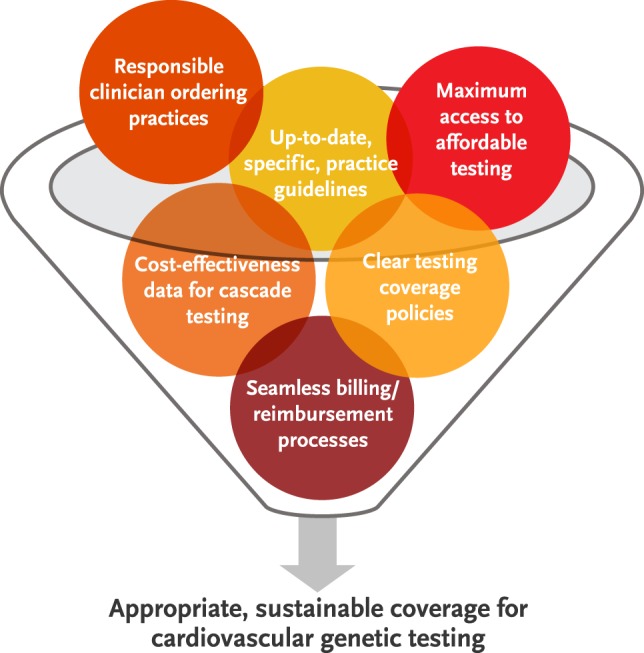
**Shared goals of clinicians, payers, genetic testing laboratories, and professional cardiology and genetics societies are starting points for the development of sustainable genetic testing practices for patients with inherited cardiovascular conditions**.

Genetic testing in the management of inherited CV conditions is here to stay. Its utility in the care of families with inherited CV conditions has been established, and genotype–phenotype correlations will likely become more refined as sequencing technologies advance. Therefore, the establishment of clear professional guidelines and consistent payer policies is crucial if affected families are to benefit from the availability of accurate and effective testing. To make recommendations and coverage work hand in hand, payers, laboratories, clinicians, and professional CV and genetics societies must collaborate and recognize their shared goals in caring for these patients (Figure 2).

Because all stakeholders agree that cascade testing can improve outcomes through early identification of individuals at risk for inherited CV conditions, the key issues are primarily those about coverage and billing. Should a family member’s insurance policy pay for CV genetic testing in a proband in some scenarios, as exemplified by the Aetna policy described above? If so, insurers must collectively determine what policy clauses or mechanisms are required to ensure that patients can benefit regardless of the coverage combinations within their families. Input from professional organizations – perhaps through more frequent updating of published CV genetic testing practice guidelines – is likely to help in standardizing the types of tests clinicians order and payers cover.

In the meantime, if billing continues to be handled by laboratories, clinicians should collect data about which tests are being covered and denied to improve responsible ordering practices. Many clinicians prefer laboratory billing because it saves time, and owing to laboratory-based customer service resources, this path often provides assurance that patients will not see unexpected bills. However, if this billing arrangement is not ensuring coverage and hides gaps in coverage, it will neither provide sustainable patient access to testing nor help clinicians advocate for changes in payer policies. Clinicians must recognize opportunities for engaging with the billing process and educating payers and guideline writers on the need for and applications of CV genetic testing.

From a health economics standpoint, available data suggest that genetic testing can provide cost savings (1, 7, 8). However, additional data are needed to demonstrate its cost-effectiveness. Also needed are specific, up-to-date practice guidelines backed by appropriate cardiology and genetics societies (e.g., HRS, ACCF, AHA, National Society of Genetic Counselors, American College of Medical Genetics and Genomics) to encourage appropriate guideline implementation and reduce misdirected use of genetic testing, which drives up health-care costs for payers without benefiting patients and families. These could be frequently re-evaluated, making any necessary updates or changes, akin to the process followed by the National Comprehensive Cancer Network for the Clinical Practice Guidelines in Oncology regarding Genetic/Familial High-Risk Assessment. Discussions between clinicians and payers about coverage will be critical as the costs of genetic testing decrease.

The current landscape of CV genetic testing is complex and involves stakeholders with different purposes, constraints, and scopes of care. No single entity can resolve the current challenges alone, and all parties must understand each other’s points of view and recognize opportunities for clearing the path toward more effective and accessible genetic testing coverage. For example, clinicians are well positioned to partner with payers to help conduct necessary research about the clinical utility of currently available CV genetic testing and cost-effectiveness of cascade screening, and professional societies are uniquely positioned to update and maintain consensus testing guidelines that can inform both clinicians and payers. Only through such collective understanding and discussion will processes and policies emerge that both safeguard clinician and patient access to testing and guarantee sustainability for laboratories and payers.

## Author Contributions

KS and NJ contributed equally to the conception of this work, the drafting and critical revision of the content, and approval of the final version to be published. They agree to be accountable for all aspects of the work in ensuring that questions related to the accuracy and integrity of any part of the work are appropriately investigated and resolved.

## Conflict of Interest Statement

KS is an employee of Indiana University, which has a clinical molecular genetics diagnostic laboratory that provides cardiovascular genetic testing. She also participated on the Invitae 2015 Cardio Advisory Board. NJ is an employee of Invitae, a commercial laboratory that provides cardiovascular genetic testing.
